# Evaluation and Calibration of CBCT Reconstruction Models

**DOI:** 10.2174/1573405619666230217121745

**Published:** 2023-05-31

**Authors:** Tao Gao, Yuchun Sun, Fusong Yuan, Shanshan Liang

**Affiliations:** 1 Dental Department, Beijing Jishuitan Hospital, Beijing, China;; 2 Center of Digital Dentistry, Department of Prosthodontics, Peking University School and Hospital of Stomatology; National Center of Stomatology; National Clinical Research Center for Oral Diseases; National Engineering Laboratory for Digital and Material Technology of Stomatology; NHC Research Center of Engineering and Technology for Digital Dentistry; Beijing Key Laboratory of Digital Stomatology, Beijing, China

**Keywords:** Accuracy evaluation, calibration, cone-beam computed tomography, model reconstruction, implantation, implant restoration

## Abstract

**Purpose:**

This study proposes a method for improving the accuracy of three-dimensional (3D) models generated through cone-beam computed tomography (CBCT).

**Methods:**

A 3D cuboid model fitted with a ¼-scale dentition on its top surface was constructed to simulate an alveolar bone with teeth. A physical specimen of the model was printed and the distance between its opposite sides was measured using a vernier caliper. The physical model was light-scanned, and the surface data of the generated 3D model were corrected by calibrating the distance between opposite sides against the vernier caliper measurements. The physical model was also scanned using CBCT to reconstruct a second 3D model. The overall deviation between the two models and the distance deviation in each direction of the cuboid and dentition were quantified and statistically analyzed.

**Results:**

The overall deviation between the reconstructed CBCT model and the calibrated structured light-scanned model was 0.098 ± 0.001 mm. Following calibration, the overall deviation was 0.010 ± 0.006 mm. A one-way variance analysis suggested that the overall deviations' differences were not statistically significant (*P* < 0.05).

**Conclusion:**

This study lays a solid foundation for accurate dental implantation.

## 1 INTRODUCTION

At present, implant restoration has become the preferred restoration and reconstruction method for most patients with dentition defects. The key to a successful implant restoration is that when placed into the alveolar bone of the edentulous area, the implant adopts a good three-dimensional (3D) position with its long axis aligned to the loading direction of the missing crown, while simultaneously avoiding critical anatomical structures such as nerves and blood vessels [1]. To correctly perform this positioning, accurate preoperative surgical planning is required, which currently relies on reconstructed 3D jaw surface models generated through computed tomography (CT) [2-4]. The reconstructed 3D models are not only used in preoperative implantation planning but also sourced to 3D-print a physical jaw model [5-7]. Together with appropriate preoperative planning, these models greatly facilitate the surgical implantation process [8]. Cone-beam CT (CBCT) has been widely implemented in multiple stomatology fields, such as preoperative implantation planning, mandibular assessment, preliminary assessment of bone mass required by orthognathic surgery, and auxiliary diagnosis and treatment of root fractures and complicated root canal surgeries [9-14]. CBCT provides satisfactory image quality that meets diagnostic needs while inducing only a low dose of radiation on patients [15-17]. However, accurate CBCT reconstruction data are essential for effective preoperative planning and intraoperative guidance. Liang *et al*. [18] reported the use of a jaw model generated with a structured light scanner as the gold standard to evaluate the accuracy of reconstructed 3D CBCT data. However, the 3D surface data acquired by structured light scanning still exhibited a certain amount of error, making it inappropriate for the accurate evaluation and calibration of CBCT results. To reliably evaluate the accuracy of CBCT reconstruction data, in this study we designed a standard evaluation method by adopting vernier caliper measurements as the gold standard, based on which we successfully evaluated and calibrated the accuracy of a reconstructed CBCT model.

## 2 MATERIALS AND METHODS

### 2.1 Definition of the Physical Specimen

**Fig. (1):**
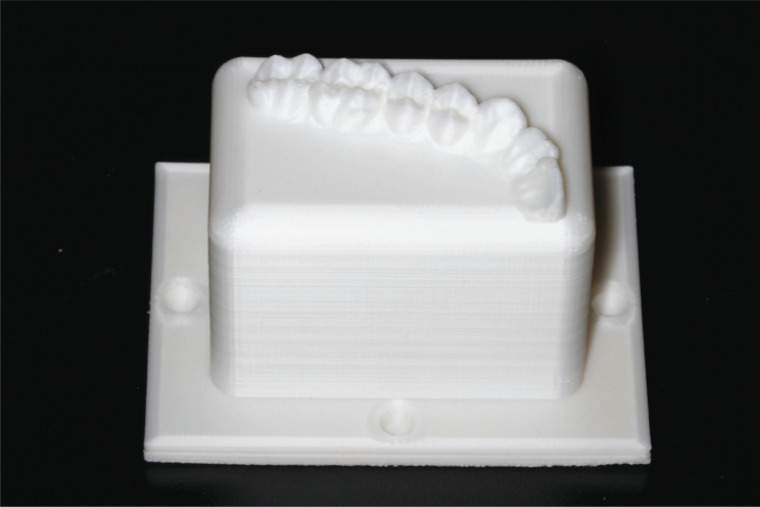
Physical standard evaluation model.

Using ImageWare (Siemens PLM Software, Berlin, Germany), a reverse engineering software, we designed a cuboid sitting on a base. At the midpoints along the four protruding edges of the base, we placed four hemispherical cavities. The top surface of the cuboid was fitted with a ¼-scale dentition, such that the cuboid itself represented the alveolar bone. The designed model was exported in STL format. Subsequently, the model was printed three times using an FDM 3D printer (Lingtong II, SHINO Tech, Beijing, China) with the slice thickness set at 0.2 mm. The dimensions of the cuboid were 60 × 40 × 40 mm (length × width × height), and those of the base were 80 × 60 × 4 mm. The diameter of the four hemispherical cavities was 5 mm. (Fig. **1**).

### 2.2 Calibration of the 3D Surface Data

The length, width, and height of the physical cuboid were measured with a vernier caliper (SATA Tools Shanghai Co., Ltd.), and then used this data as the gold standard. Subsequently, it was imported into Geomagic Studio 12.0 (Raindrop, Morrisville, NC, USA) to measure the corresponding digital model. And the scaling ratio of the length, width, and height was calculated, then the scaling function in Geomagic was used to scale the digital model to complete the calibration. Finally, the three-dimensional surface data obtained by the scaled structured light scanner was compared with the data measured with the vernier caliper to minimize the error.

### 2.3 Acquisition of CBCT Data and Reconstruction of the Physical Specimen Surface Model

**Fig. (2):**
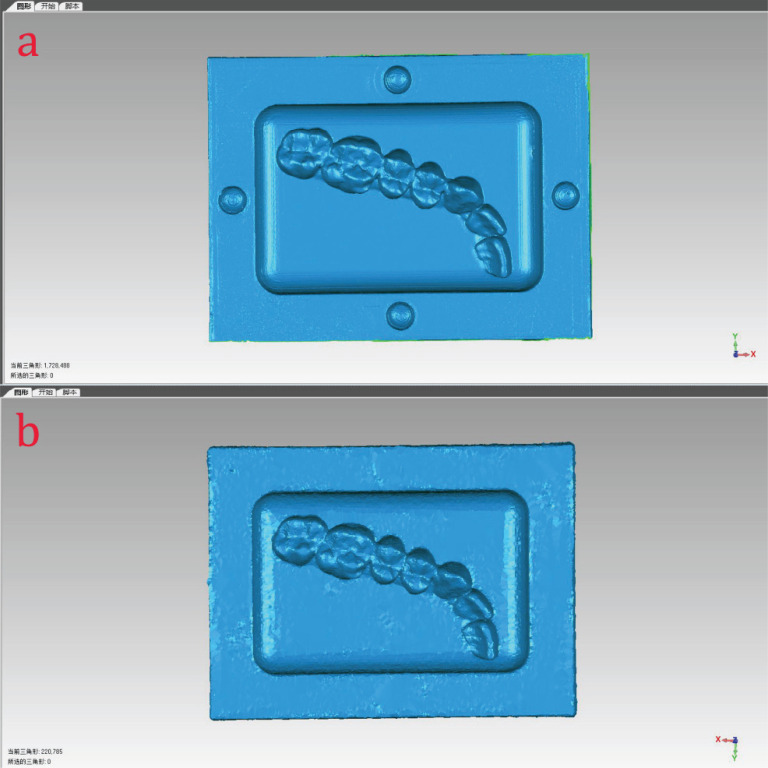
Structured light scanned model (**a**) and reconstructed CBCT model (**b**).

After filling the four hemispherical cavities with gutta-percha, the physical specimen was placed in a plastic container and immersed in water to simulate the oral cavity environment. Subsequently, the model was scanned using a CBCT (New Tom VG, Verona, Italy), and the scanned data were exported as DICOM. Once the 3D data of the evaluation model were constructed in mimics (Mimics 17.0, Leuven, Belgium), they were exported in STL format and labeled as DATA1 (Fig. **2a**). Then, using a structured light scanner (Activity 880; Smart Optics, Bochum, Germany), 3D surface data of the specimen were also acquired, exported in STL format, and labeled as DATA2 (Fig. **2b**).

### 2.4 Registration of the Reconstructed 3D CBCT and 3D Light Scanner Surface Datasets

**Fig. (3):**
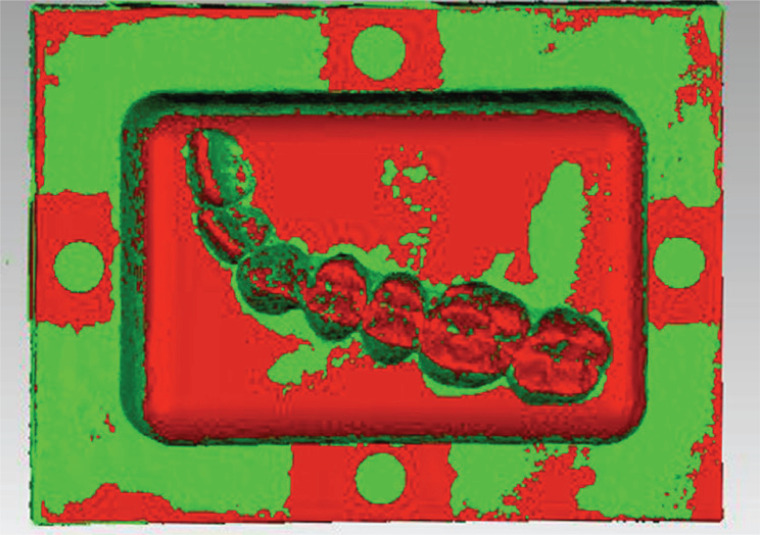
Registration result of the two 3D datasets. The distance difference represented by the green color is smaller than the red one.

After the reconstructed 3D datasets recorded through CBCT and light scanning were both imported into the Geomagic software, the central points of the four hemispherical cavities on the base of the reconstructed models were identified. Subsequently, the two datasets were co-registered to the same coordinate system through registration based on the four central points, global registration, and best-fitting registration (Fig. **3**).

### 2.5 Quantification and Statistical Analysis of the Deviations between the Two Datasets

**Fig. (4):**
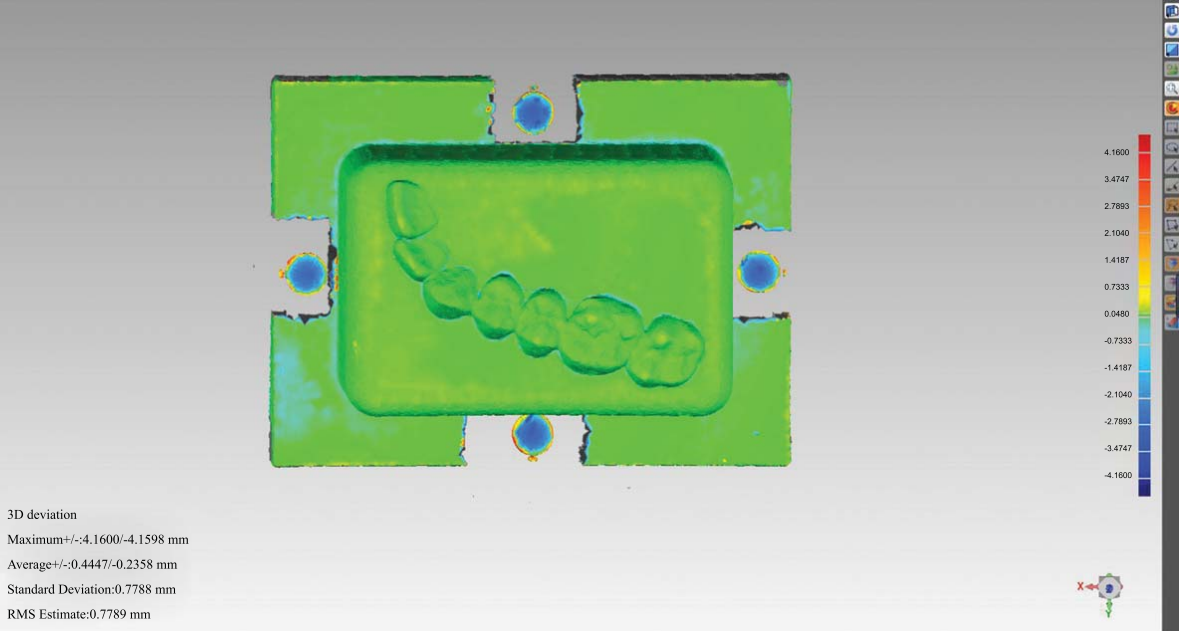
Measurement of overall deviation of the two 3D datasets.

The overall deviation as well as the individual deviations between the height, width, and length of the two datasets were quantified and statistically analyzed using Geomagic. In addition, the same comparison and analysis were repeated after the calibration of the reconstructed CBCT data (Fig. **4**).

## 3 RESULTS

Compared with the vernier caliper measurements, the deviations in the length, width, and height of the 3D data acquired using the structured light scanner were 0.034 ± 0.006, 0.029 ± 0.011, and 0.020 ± 0.003 mm, respectively (Table **1**). After calibration, these deviations decreased to 0.008 ± 0.002, 0.008 ± 0.003, and 0.003 ± 0.002 mm, respectively (Table **2**). Moreover, when comparing the reconstructed CBCT dataset with the calibrated structured light scanned dataset, the overall deviation of the entire cuboid was 0.098 ± 0.001 mm, the overall deviation of the dentition was 0.089 ± 0.002 mm, and the deviations in length, width, and height were 0.213 ± 0.029, 0.311 ± 0.028, and 0.031 ± 0.006 mm. Following the calibration of the CBCT data, the overall deviation of the entire cuboid was reduced to 0.010 ± 0.006 mm, the overall deviation of the dentition was decreased to 0.006 ± 0.002 mm, and deviations in length, width, and height became 0.055 ± 0.021, 0.014 ± 0.013, and 0.007 ± 0.008 mm. A one-way analysis of variance (ANOVA) suggested that the differences between the deviations for the entire model, dentition, and length, width, and height were not statistically significant (P < 0.05).

## 4 DISCUSSION

Tis study aimed to establish a standard evaluation model to assess the accuracy of reconstructed 3D CBCT models and improve their geometric accuracy *via* calibration. The results indicated that, when compared with vernier caliper measurements, the 3D data acquired using structured light scanning still exhibited a certain amount of error. However, software calibration could substantially improve the accuracy of the reconstructed data. In addition, compared with the 3D data from the structured light scanner, data acquired through CBCT demonstrated a considerably lower accuracy, most likely owing to the high level of noise in the two-dimensional axial slices of CBCT, particularly when the slices are thin. Several artifacts inherent to CBCT image acquisition and associated with detector sensitivity, X-ray beam nonuniformity, and reconstruction algorithms [19, 20] contributed to this noise. The combined effect significantly increases the noise level, thereby affecting the accuracy of the reconstructed 3D surface model. Furthermore, appropriate thresholds need to be established during 3D CBCT acquisition to block soft tissue and clearly display bone tissue and enamel. However, these thresholds depend on multiple factors, such as the patient’s bone density, scanning dose, and physician’s experience. Patients with large bone densities require higher thresholds, and *vice versa*. In addition, soft tissue thickness and bone structure heterogeneity can weaken the electromagnetic waves to varying degrees. Finally, the 3D model reconstruction of CBCT is realized by displaying the datasets on the computer using different display technologies. Since human eyes recognize the 3D model by viewing light and shade on the screen, errors may be introduced when locating a point. However, compared with structured light scanning, CBCT still presents distinctive advantages. For example, the multi-view reconstruction function of CBCT can accurately evaluate the height, width, and cortical bone thickness of the alveolar ridge, as well as the positions of the nasopalatine canal, maxillary sinus floor, and septum, so that the implant can receive maximum possible bone support after establishment [21].

In this study, we improved the accuracy of the model registration process by adopting an automatic surface-matching algorithm based on the registration of characteristic surface points together with global and best-fitting registration. The main advantage of this method is that the entire 3D surface was used for the registration, instead of individual characteristics. In addition, this process reduced the impact of errors introduced by manual operations on measurement accuracy [22].

A previous study [23] reported that CT scanning a dry object in the open air would produce artifacts. Therefore, in this study CBCT scans of an oral prosthesis were performed after the container was filled with water, which not only simulated the humid environment of the oral cavity but also prevented possible artifacts caused by exposing the prosthesis to dry air. Consequently, the resultant images were more realistic. In actual clinical implementations, metal prostheses or amalgam fillings in the patient’s mouth may introduce artifacts in CBCT images, thereby affecting the segmentation accuracy [24, 25]. As a result, further research on the reduction and compensation of CBCT artifacts is needed.

## CONCLUSION

Although the reconstruction accuracy of CBCT is lower than that of structured light scanning, it was substantially improved to a level that satisfies clinical needs after calibration. Therefore, CBCT provides the necessary preconditions for accurate clinical implantation. The proposed method applies to various types of CBCT systems. Therefore, future research will focus on the verification of each.

## Figures and Tables

**Table 1:** Deviation between structured light scanned 3D data and vernier caliper measurement before and after calibration.

-	**Length Deviation (mm)**		**Width Deviation (mm)**		**Height Deviation (mm)**	
	**Before Calibration**	**After Calibration**	**Before Calibration**	**After Calibration**	**Before Calibration**	**After Calibration**
**Model 1**	0.039	0.007	0.020	0.011	0.019	0.002
**Model 2**	0.027	0.010	0.025	0.005	0.024	0.003
**Model 3**	0.036	0.006	0.041	0.007	0.018	0.005
**Mean ± SD**	0.034 ± 0.006	0.008 ± 0.002	0.029 ± 0.011	0.008 ± 0.003	0.020 ± 0.003	0.003 ± 0.002

**Table 2:** Deviation between CBCT scan reconstruction data and structured light scanned data before and after calibration.

**-**	**Overall Deviation** **(mm)**		**Dentition Deviation** **(mm)**		**Length Deviation** **(mm)**		**Width Deviation** **(mm)**		**Height Deviation** **(mm)**	
	**Before Calibration**	**After Calibration**	**Before Calibration**	**After Calibration**	**Before Calibration**	**After Calibration**	**Before Calibration**	**After Calibration**	**Before Calibration**	**After ** **Calibration**
Model 1	0.097	0.0058	0.091	0.0045	0.181	0.0361	0.321	0.0058	0.026	0.0050
Model 2	0.097	0.0078	0.087	0.0056	0.238	0.0518	0.279	0.0060	0.038	0.0058
Model 3	0.100	0.0173	0.090	0.0085	0.219	0.0781	0.334	0.0287	0.030	0.0090
Mean ± SD	0.098 ± 0.001	0.010 ± 0.006	0.089 ± 0.002	0.006 ± 0.002	0.213 ± 0.029	0.055 ± 0.021	0.311 ± 0.028	0.014 ± 0.013	0.031 ± 0.006	0.007 ± 0.008

## Data Availability

The data presented in this study are available on request from the corresponding author [Y.S].

## LIST OF ABBREVIATIONS

CBCT: Cone-beam Computed Tomography
CT: Computed Tomography
